# Genetically assembled fluorescent biosensor for *in situ* detection of bio-synthesized alkanes

**DOI:** 10.1038/srep10907

**Published:** 2015-06-03

**Authors:** Wei Wu, Lei Zhang, Lun Yao, Xiaoming Tan, Xufeng Liu, Xuefeng Lu

**Affiliations:** 1 Key Laboratory of Biofuels, Shandong Provincial Key Laboratory of Energy Genetics, Qingdao Institute of Bioenergy and Bioprocess Technology, Chinese Academy of Sciences, Qingdao, China; 2 University of Chinese Academy of Sciences, Beijing, China

## Abstract

Construction of highly efficient microbial cell factories producing drop-in biofuel alkanes is severely limited due to the lack of a fast detection method against alkanes. Here we first developed a sensitive fluorescent biosensor for rapid and *in situ* monitoring of intracellular alkane synthesis. Using GFP as reporter, the biosensor could actively respond to the intracellular alkane products, especially for the mid- and long-chain alkanes synthesized in the recombinant *Escherichia coli* and give a concentration-dependent fluorescence response. Our results also suggested the feasibility of developing high-throughput strategies basing on the alkane biosensor device in *E. coli*, and thus will greatly facilitate the application of directed evolution strategies to further improve the alkane-producing microbial cell factories.

Producing aliphatic hydrocarbons using microorganisms, which can be used as advanced biofuels, has been an attractive idea since the discovery of the natural alkane-producing species. In recent years, several artificial alkane/alkene-producing systems have been constructed by re-assembling the natural alkane-biosynthesizing pathways consisting of, for example, a cyanobacterial acyl-ACP reductase (AAR) and an aldehyde-deformylating oxygenase (ADO) in the platform strains such as *Escherichia coli*, leading to synthesis of medium- and long-chain alkanes (C9-C17)^1^,^2^. However, the yield has been low, and a few studies have reported increasing alkane production by redirection of the carbon flux or try-out of other alkane synthesis pathways^2^,^3^,^4^,^5^,^6^. Nevertheless, further evolution of the cell factories and key enzymes for higher alkane productivity is still severely limited because of the lack of fast and high-throughput intracellular alkane detection methods. Thus it is urgent to develop a rapid and generic alkane detection method by which the accumulation of biosynthesized alkanes in the cell could be sensitively and conveniently probed^7^. At present, detection of alkanes is still a challenge due to the relatively inert property and the lack of characteristic functional groups in the molecules, and as a result gas chromatography (GC) and mass spectrometry (MS) have been the few methods available for the quantification of intracellular alkanes thus far, which are however costly and time-consuming.

Interestingly, in some natural bacteria such as *Acinetobacter* sp. and *Pseudomonas* sp^8^,^9^, alkanes act as carbon source and also as signal molecules in the alkane degradation pathways. Those bacteria can take in alkanes from outside of the cell and initiate the expression of downstream genes of which the expression rate rests with the environmental alkane concentration. The alkane responsive transcriptional regulatory proteins such as AlkR in *A. baylyi* ADP1^9^ or AlkS in *P. oleovorans*^10^, which can recognize alkanes and activate the respective promoter P_*alkM*_ or P_*alkB*_ in the presence of alkanes, are playing important roles in these processes. Such a phenomenon sheds light on the rapid detection of alkanes. For example, fluorescent bacterial alkane biosensors AlkR-P_*alkM*_ and AlkS-P_*alkB*_ have been applied as useful bacterial biosensor plugins to the environmental alkane detection^8^,^11^ as well as to the monitoring of microbial alkane uptake^12^. However, at present there has not yet been successful examples of *in situ* probing the endogenous alkanes (especially medium- and long-chain alkanes) in a recombinant alkane-producing strain using similar biosensors, which is most likely due to the limitation of the substrate specificity as well as the host specificity of the two most used biosensors mentioned above. A previous study has shown that the *Acinetobacter* ADP1- originated biosensor AlkR-P_*alkM*_, which has a natively wide alkane detection spectra in *Acinetobacter*^11^, could not work when harbored in *E. coli* (Schirmer, A., *et al*., US Patent 2008/0293060 A1). Another alkane biosensor AlkS-P_*alkB*_ from *Pseudomonas* can only respond to alkanes of short and medium carbon chains (*i.e.*, C5–C11) in the *E. coli* hosts^8^,^13^.

As long-chain alkanes (C13-C17) were usually the main products due to the relatively abundant intracellular long-chain fatty acids (C14–C18) in *E. coli*^2^, it was important to develop a new alkane biosensor which was especially responsive to the long-chain alkanes in *E. coli*. To achieve this purpose, we reassembled the two natural alkane-responsive plugins AlkR-P_*alkM*_ and AlkR-P_*alkM*_ and developed a synthetic chimera alkane response element (cARE) in *E. coli* with green fluorescent protein (GFP) as reporter. We transformed the recombinant alkane producing *E. coli* with cARE, and alkane concentration-dependent fluorescence responses induced by the endogenous alkanes could be observed, providing a visualized and high-throughput way of monitoring the alkane synthesis in the recombinant *E. coli*.

## Results

### Genetic construction of the synthetic chimera alkane response element (cARE) and expression in *E. coli*

**Figure 1:**
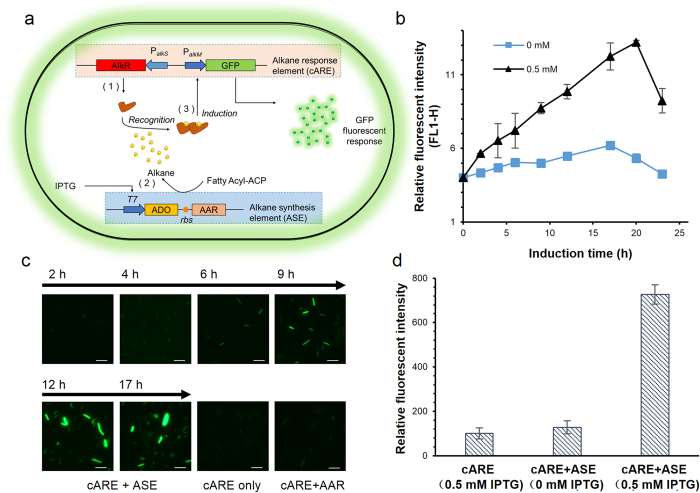
Intracellular alkane biosensor construction and functional characterization. (**a**) Schematic for the *in situ* probing of endogenous alkane in *E. coli* basing on the synthetic alkane biosensor cARE. (**b**) Dynamic cARE response curve of the strains harboring both cARE and ASE under 0 mM (■) or 0.5 mM (▲) of IPTG. Increasing fluorescence intensities of the IPTG induced cells along with the induction time after induction were shown using a flow cytometer machine via the FL1-H channel (with excitation 488 nm, emission 515–535 nm). The mean values of fluorescence intensities of the sampled cells were determined. Error bars gave the means of parallel tests. (**c**) Visualized response of cARE to alkane in recombinant *E. coli* cells by fluorescence microscopy under 0.5 mM IPTG induction. The images of the negative controls at 17 h (cARE only and cARE+AAR) were shown. (**d**) The relative fluorescent intensity of the culture 17 h after induction using fluorescence microplate reader (emission at 475–495 nm, excitation at 506–526 nm). Error bars gave the means of three independent cultures for each strain. cARE, the chimera alkane response element; ASE, alkane synthesis element; ADO, aldehyde-deformylating oxygenase; AAR, acyl-ACP reductase; GFP, green fluorescent protein; FL1-H, channel for GFP fluorescence assay with 488 nm emission and 515–545 nm excitation)

Generally, to construct an alkane biosensor element, three units were necessary, including an alkane-responsive regulator protein, the promoter that was controlled by the regulator, and a reporter protein (*e.g*. GFP in this work) driven by the promoter. As reported previously, AlkR-P_*alkM*_ from *Acinetobacter* natively had a wide alkane detection spectra (especially for the medium- and long- chain alkanes)^11^, thus was a good candidate for monitoring the intracellular alkane synthesis in *E. coli*. In fact, the idea that using the AlkR-P_*alkM*_ biosensor in *E. coli* was not new. However, almost no successful implementations had yet been reported except a recent patent specification (Schirmer, A., *et al*., US Patent 2008/0293060 A1) where it was mentioned that even when the regulator protein AlkR was successfully expressed in *E. coli* when under an isopropyl *β*-d-1-thiogalactopyranoside (IPTG) inducible promoter, the AlkR-P_*alkM*_ biosensor alone was still insufficient for initiating the alkane-induced expression of reporter gene. One conjecture was made that there might be some additional gene necessary for the alkane detection in the heterologous hosts. Anyhow, to avoid this issue here, taking into consideration of the good efficacy of AlkS-P_*alkB*_ in *E. coli*, we decided to reassemble the two elements AlkR-P_*alkM*_ and AlkS-P_*alkB*_ and constructed a chimera one (cARE) (Fig. 1a) in which the constitutive promoter P_*alkS*_ from AlkS-P_*alkB*_ was used instead to drive the expression of transcriptional regulator AlkR, and AlkR then activated the promoter P_*alkM*_ and initiated GFP expression in the presence of alkanes. The cARE gene was cloned into the previously constructed platform strain *E. coli* BL21(DE3)Δ*fad*E, in which the deletion of the *fadE* gene encoding acyl-CoA dehydrogenase could enhance the accumulation of fatty acyl-CoA as well as the downstream metabolites such as alkanes if the alkane biosynthesis pathway existed^14^. To verify the expression of AlkR protein in *E. coli*, we alternatively attached a hexahistidine tag to the C-terminus of AlkR in the cARE and succeed in probing the 35 kDa soluble target protein (AlkR) from the cell extracts using an anti-polyhistidine antibody (Supplementary Fig. 1), demonstrating that the P_*alkS*_-driven AlkR could be constitutively expressed in *E. coli*.

### *In situ* alkane detection by cARE in the alkane producing strain

We had proved that AlkR could be constitutively expressed in *E. coli* by the new promoter. To verify whether the AlkR could respond to alkanes in this system, an attempt of adding heptadecane (C17) from outside of the *E. coli* cells were carried out but it failed to lead to the expression of GFP (Supplementary Fig. 2), probably due to the extremely low solubility and accessibility of C17^11^,^12^. To avoid the possible transfer problems, we then integrated the alkane synthesis element (ASE) into the host strain which already harbored the cARE plasmids. The alkane synthesis element consisted of a T7 promoter, the open reading frames orf1593 (ADO) and orf1594 (AAR) from *Synechococcus elongatus* PCC7942 (Fig. 1a). In particular, alkane synthesis was initiated by adding 0.5 mM of IPTG. We monitored the average fluorescence intensities of the cell suspensions by flow-cytometric analysis (FL1-H, channel for GFP, with 488 nm excitation and 515–545 nm emission) (Fig. 1b), acquired the fluorescence images of the cells by fluorescence microscopy (Fig. 1c) and quantitated the relative fluorescent intensity using a fluorescent microplate reader (with 475–495 nm excitation and 506–526 nm emission) (Fig. 1d). As observed, cARE was rapidly activated and expressed GFP within 6 h, and a maximum 6-fold increase of green fluorescence compared to that under the non-induced condition in absence of IPTG was observed after approximately 17 h of incubation (Fig. 1d). The alkane products (mainly pentadecane and heptadecene) extracted from the cell extracts could also be detected by GC-MS (Supplementary Fig. 3). These results indicated that the expression of the alkane synthesis pathway could apparently initiate the expression of reporter gene in cARE. Furthermore, to rule out the possibility that the activation of P_*alkM*_ promoter and GFP expression was due to IPTG itself as well as the possible interfering species generated by AAR, we also evaluate the background fluorescence in the non-alkane producing *E. coli* BL21(DE3)Δ*fad*E variants harboring either only cARE (Fig. 1c, cARE) or both cARE and AAR (Fig. 1c, cARE+AAR), as negative controls (Supplementary Fig. 4). Indeed, only a background level of fluorescence was observed compared with the strains co-harboring the biosensor and AAR-ADO pathway (Fig. 1c, cARE+ASE). Thus, we could deduce that cARE actively responded to the alkane products (pentadecane and heptadecene) generated from ADO.

### Alkane yields-dependent fluorescence response of cARE in *E. coli*

**Figure 2:**
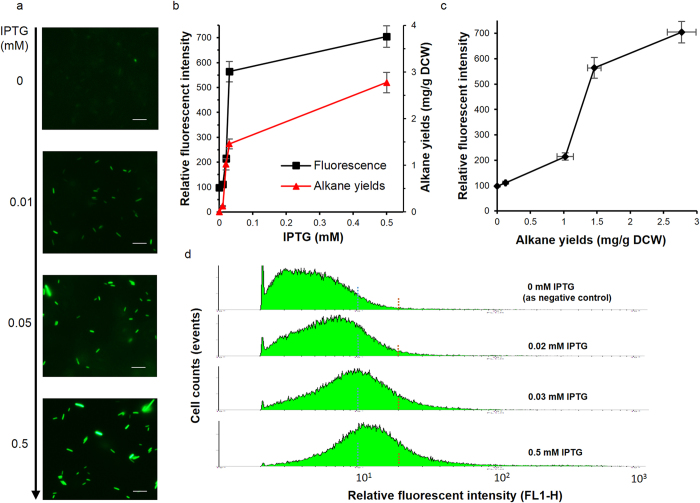
Fluorescence response of cARE and alkane production in recombinant E. coli under different induction level. (**a**) Fluorescence imaging of alkane-producing *E. coli* harboring cARE in presence of 0 mM, 0.01 mM, 0.05 mM or 0.5 mM IPTG in 17 h after induction. (**b**) The fluorescent responses (■) and the final alkane yields (▲, mainly pentadecane and heptadecene) under different IPTG concentration; (**c**) Plotted curve of fluorescence response against alkane productivity under different IPTG concentrations (emission 475–495 nm, excitation 506–526 nm). Error bars gave the means of three independent cultures for each strain. (**d**) Fluorescence activated cell analysis of cells under different IPTG concentration by a flow cytometer machine. The blue dashed lines showed the fluorescent threshold of the top 10% of the control cells (0 mM IPTG), and the red dashed lines showed the fluorescent threshold of the top 1% of the control cells. FL1-H, channel for GFP fluorescence assay (emission at 488 nm, excitation at 515–545 nm).

To evaluate the correlation between the fluorescent responses and alkane yields, the strains harboring both alkane synthesis element (ASE) and the detection element (cARE) were induced by different level of IPTG from 0 mM to 0.5 mM (Fig. 2a). The relative fluorescence responses of the strains were visually and quantitatively determined. Obvious fluorescence enhancement could be observed under fluorescent microscopy, and a saturation-type dependency of fluorescence responses on IPTG concentrations was clearly shown. Using a microplate reader to quantitate the fluorescent intensity, (Fig. 2b), in which a nearly 6 times enhancement of fluorescence for the cells induced by 0.5 mM IPTG was observed than the negative control, and the apparently lowest IPTG concentration for the alkane-induced GFP expression was about 0.02 mM in this system. Meanwhile, the alkane yields of the strains under different level of IPTG induction were also determined with GC-MS using n-eicosane (C20) as internal standard^5^. A similar saturation-type dependency of alkane yields on IPTG concentrations was similarly shown, with alkane yields ranging from 0 to 4.56 ± 0.56 mg/L culture (2.77 ± 0.22 mg/g dry cell weight, DCW) (Fig. 2b). It was also noticed that 0.02 mM IPTG was enough to induce obvious alkane synthesis (1.85 ± 0.51 mg/L culture, 1.02 ± 0.12 mg/g DCW), which was about 30% of the alkane yields under 0.5 mM IPTG. Eventually, a typical “S-curve” type correlation between the fluorescence responses and the alkane yields was presented by plotting the relative fluorescent intensity and respective alkane yields (Fig. 2c).

### Exploring the possibilities of developing high-throughput strategies basing on flow cytometry

To demonstrate the feasibility of the alkane biosensor for high-throughput screening of the alkane producing cells, we then analyzed the fluorescent intensity distributions of cells in presence of varying IPTG concentration (0, 0.02 mM, 0.03 mM, 0.5 mM, 17 h after induction) using a flow cytometer. With the IPTG concentration raised, a clear tendency of increasing and enhancing was shown for the fluorescent cells (Fig. 2d). In particular, if setting the fluorescent intensity to be the top 1% of the control strain as the fluorescence threshold level, more than 11% cells induced by 0.5 mM IPTG could be distinguished (5% cells for 0.03 mM IPTG and 2% cells for 0.02 mM IPTG, respectively), which roughly meant a theoretical positive rate of about 90% in that fluorescence region when screening alkane producing strains from the library harboring the potential alkane synthesis processes as well as the alkane detection plugins cARE. Besides, while there were scarcely any cells with FL1-H >10^2^ in the control strains, there were still considerable amounts of cells (nearly 1% of the total cells) with FL1-H >10^2^ in the strains that induced by 0.5 mM IPTG. This increasing cell amount in the relatively stronger fluorescence region implied that we might be able to simply and efficiently separate the cells with higher alkane productivity from a library consisting of billions of cells with a relatively higher fluorescence threshold.

## Discussions

Construction of highly efficient microbial cell factories producing drop-in biofuel alkanes was an important issue for biofuel production, and developing a simple, convenient and high-throughput method to fast evaluate or estimate the alkane production level in the cells was one of the necessary technical foundations which would greatly facilitate the exploring of alkane-producing enzymes and biosynthesis pathways. In this work we first described the development of a genetically modified and assembled alkane biosensor to *in situ* evaluate the alkane production in a widely used recombinant platform strain *E. coli*. With GFP as reporter gene, the relative alkane production level of the cells could be roughly estimated according to the relative green fluorescent intensity.

Our results showed that the *in situ* biosensor was capable of sensing alkane yields roughly as low as about 1 mg/g DCW. With IPTG concentration raised from 0 to 0.5 mM, the increase of alkane yields led to enhancement of fluorescence response. We also noticed that the correlation between the fluorescence responses with the alkane presented was somehow partly similar to the previously reported response curve of *Acinetobacter* biosensor (ADPWH_alk) AlkR-P_*alkM*_ to crude oil in soil^11^. Our results implied that the synthetic element cARE could be used to roughly estimate the *in vivo* alkane production level in the alkane-producing *E. coli*, which would greatly facilitate the further studies in the related fields.

An interesting phenomenon we noticed was the enormous variation in the fluorescent intensity of the cells under the fluorescence microscopy (Figs. 1c,2a), which was even more clearly presented in the flow cytometric results (Fig. 2d). According to the cell counts distributions versus the FL1-H value (the relative fluorescent intensity of GFP, 515 nm–545 nm), while over 90% of the non-induced cells (Fig. 2d, 0 mM) were of negligible or weak fluorescence (FL1-H < 10, for example), there were still very few cells of obvious or even strong fluorescence. On the other hand, whereas the increase of IPTG led to gradually increasing amounts of the stronger fluorescent cells, there were still about 50% cells with weak fluorescence that FL-H < 10 even under 0.5 mM IPTG. This result implied that at least either the alkane response or the alkane synthesis was not homogeneous but of enormous diversity at the single cell level, and thus the relative fluorescence intensities by microplate readers were only the average values of the cells which were however increasing with the inducer concentration. The reasons for this fluorescence heterogeneity was not clear so far. One possible reason might be the probably heterogeneous distribution of the very low contents of long-chain alkane products in the single cells (less that 1% of DCW) due to its extremely low solubility in physiological environment. In fact, the cytoplasmic distribution of the alkane products in *E. coli* was also waiting for answer. Anyhow, this variation of the fluorescent intensity at the single cell level might cause some undesirable false positives when screening mutants from a large library. Even though, taking into consideration of good consistence between the mean fluorescent fluorescence and alkane yields, as well as the dramatically increasing numbers of the cells with stronger fluorescence under higher induction level, the screening of cells with higher alkane productivity would still benefit from the fluorescence activated cell sorting (FACS) technology in which higher fluorescence threshold would largely decrease the rate of false positives.

In conclusion, we have first provided a convenient alkane biosensor cARE for rapid and *in situ* detection of intracellular alkanes synthesized by recombinant *E. coli*, especially for detection of medium- and long- chain alkanes in *E. coli*. Though further optimization for the biosensor are still needed to increase the fluorescence response and to reduce the diversity of fluorescent signals at the single-cell level, the biosensor presented in this work has enabled the high-throughput evaluation of the alkane production in an alkane-producing strains for the first time. We also believe that our work will largely promote the construction of highly efficient alkane-producing microbial cell factories.

## Methods

### Construction of the biosensors

We amplified the DNA sequences of AlkR and P_*alkM*_ from *Acinetobacter baylyi* ADP1 genome (as a gift from Prof. Huang’s lab) using the primers alkR-F/alkR-R (Supplementary Table 1) or alkR-F/alkR-his-R and alkM-F/alkM-R, respectively; we amplified P_*alkS*_ using the primers alkS-F/alkS-R from the plasmid pCom8^15^ (GenBank: AJ299427.1, as a gift from Prof. Hang’s lab) with an EGFP gene insertion between the *Eco*RI and *Sal*I sites of the plasmid. The three DNA segments were then assembled by overlapping PCR to generate 1.3 kB DNA product which was then subcloned into pCom8-EGFP between the *Xho*I and *EcoR*I sites.

### Assembling the sensor and alkane biosynthesis pathway in *E. coli*

We amplified the DNA sequence encoding both ADO (*Synechococcus elongatus* PCC7942 orf1593, YP_400610) and AAR (*S*. *elongatus* PCC7942 orf1594, YP400611) from the genome of *S*. *elongatus* PCC7942 using the primers 1593-F and 1594-R and cloned it into the *Nde*I and *Xho*I sites of pET21b(+) (Novagen) under the control of the T7 promoter. We also constructed another pET21b vector harboring only AAR (orf1594) between the *Nde*I and *Xho*I sites. The host strain *E. coli* BL21(DE3)Δ*fad*E with deletion of the *fadE* gene encoding acyl-CoA dehydrogenase was constructed in accordance with previous studies^14^ and was either transformed to harbor the alkane biosensor plasmid cAREp alone or co-transformed to harbor both cAREp and AAR-ADO alkane biosynthesis pathway. As a control, BL21(DE3)Δ*fad*E harboring both cAREp and AAR was also constructed. For western blotting detection, the cells harboring cARE were incubated at 30 °C for 40 h in LB medium (supplemented with 50 mg/L gentamicin) before cell disruption. The soluble fractions and insoluble fractions of the cell extracts were separated by 12% SDS-PAGE according to a standard procedure and electroblotted onto PVDF membranes, sealed in 5% nonfat milk-PBST (0.05% Tween-20 in 10 mM phosphate buffer saline) at room temperature for 1 h. In detail, the membranes were first incubated with the anti-His primary antibodies for 3 h and washed three times with PBST and then incubated with a Horseradish Peroxidase-linked secondary antibody for 1 h and washed three times with PBST and finally colored using a HRP-DAB Coloration Kit (TIANGEN, China).

### Alkane synthesizing and biosensor induction in *E. coli*

The recombinant cells were grown in the modified mineral medium^1^ (containing 6 g/L Na_2_HPO_4_, 3 g/L KH_2_PO_4_, 0.5 g/L NaCl, 2 g/L NH_4_Cl, 0.25 g/L MgSO_4_·7H_2_O, 11 mg/L CaCl_2_, 27 mg/L FeCl_3_·6H_2_O, 2 mg/L ZnCl_2_·4H_2_O, 2 mg/L Na_2_MoO_4_·2H_2_O, 1.9 mg/L CuSO_4_·5H_2_O, 0.5 mg/L H_3_BO_3_, 1 mg/L thiamine, 200 mM Bis-Tris (pH 7.25) and 0.1% v/v Triton-X100, with 30 g/L glucose as carbon source) supplemented with 50 mg/L gentamicin (for strains harboring cAREp only) or 50 mg/L gentamicin and 100 mg/L ampicillin (for strains harboring dual plasmids). Isopropyl *β*-d-1-thiogalactopyranoside (IPTG) was added to make a final concentration ranging from 0 mM to 0.5 mM to induce the enzyme expression and initiate the alkane synthesis at an OD_600_ of 0.4 at 30 °C. The cells were incubated for another 17 h before fluorescent analysis and imaging. Non-alkane-producing recombinant variants harboring either only cAREp or both cAREp and AAR were also incubated with IPTG as controls.

### Alkane extraction and GC/MS analysis

To evaluate the total alkane yields (including alkanes and alkenes), cultures were incubated for another 40 h since IPTG induction at 30 °C. The dry cell weights (DCWs) were determined; and another 10 ml of cultures were harvested and extracted with 10 ml of chloroform–methanol (v/v, 2:1). The organic phase was concentrated by evaporation, dissolved in n-hexane and finally analyzed by GC/MS using an Agilent 7890A-5975C system equipped with a HP-INNOWax (30 m × 250 μm × 0.25 μm). Helium (constant flow 1 mL/min) was used as the carrier gas. The temperature of the injector was 250 °C and the following temperature program was applied: 100 °C for 1 min, increase of 5 °C min^−1^ to 150 °C then increase of 10 °C min^−1^ to 250 °C for 15 min. To evaluate the alkane yield, *n*-eicosane (Sigma-Aldrich) was used as an internal standard, which was added to the culture before extraction. The alkane yields were calculated according to the peak areas of the respective hydrocarbons in the GC spectrum.

### Fluorescent quantification, imaging and fluorescence activated cell analysis

We measured the fluorescence of the cell suspensions with a Synergy^HT^ fluorescence microplate reader (BioTek): excitation wavelength was 485 ± 10 nm; emission wavelength was 516 ± 10 nm. The relative fluorescent intensity (RFI) was calculated (GFP emission/OD) and normalized to the intensity of cells harboring cARE. Fluorescence images of the cells were taken with an Olympus BX51 fluorescence microscopy equipped with an Olympus U-RFL-T mercury burner. To perform fluorescence activated cell analysis, cells were centrifuged and resuspended with 0.01 mM PBS to a density of approximately 10^6^ cells/ml and analyzed using a BD FACSCalibur™ machine via FL1-H channel (with excitation 488 nm, emission 515–545 nm). The total amount of cells gated for analysis was 50,000.

## Additional Information

**How to cite this article**: Wu, W. *et al*. Genetically assembled fluorescent biosensor for *in situ* detection of bio-synthesized alkanes. *Sci. Rep*. **5**, 10907; doi: 10.1038/srep10907 (2015).

## Supplementary Material

Supplementary Information
