# Supplementary material for: Genetically assembled fluorescent biosensor for in situ detection of bio-synthesized alkanes
Source: Sci Rep. 2015 Jun 3;5:10907. doi: 10.1038/srep10907 (PMC5387116; doi:10.1038/srep10907)
Supplement: Supplementary Information [file srep10907-s1.pdf]

Supplementary Information

Genetically assembled fluorescent biosensor for *in situ* detection of bio-synthesized alkanes

Wei Wu[1#], Lei Zhang[1,2#], Lun Yao[1,2], Xiaoming Tan[1], Xufeng Liu[1,2], Xuefeng Lu[1*]

[1]Key Laboratory of Biofuels, Shandong Provincial Key Laboratory of Energy Genetics, Qingdao Institute of Bioenergy and Bioprocess Technology, Chinese Academy of Sciences, Qingdao, China. [2]University of Chinese Academy of Sciences, Beijing, China.

[#] These authors contributed equally to this work.

Correspondence and requests for materials should be addressed to X.L. (Lvxf@qibebt.ac.cn).

Supplementary Fig. 1

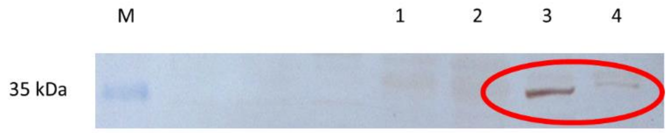

Supplementary Fig. 1| Detection of AlkR expression by western blotting using an anti-polyhistidine antibody. The size of AlkR was in agreement with the theoretical value (35.6 kDa, as marked in Lane 3). Lane 1, soluble fraction of BL21(DE3)*ΔfadE* as control; lane 2, insoluble fraction of BL21(DE3)*ΔfadE* as control; lane 3, soluble fraction of BL21(DE3)*ΔfadE* harboring cARE; lane 4, insoluble fraction of BL21(DE3)*ΔfadE* harboring cARE. Cells were incubated for 40 h before harvesting.

Supplementary Fig. 2

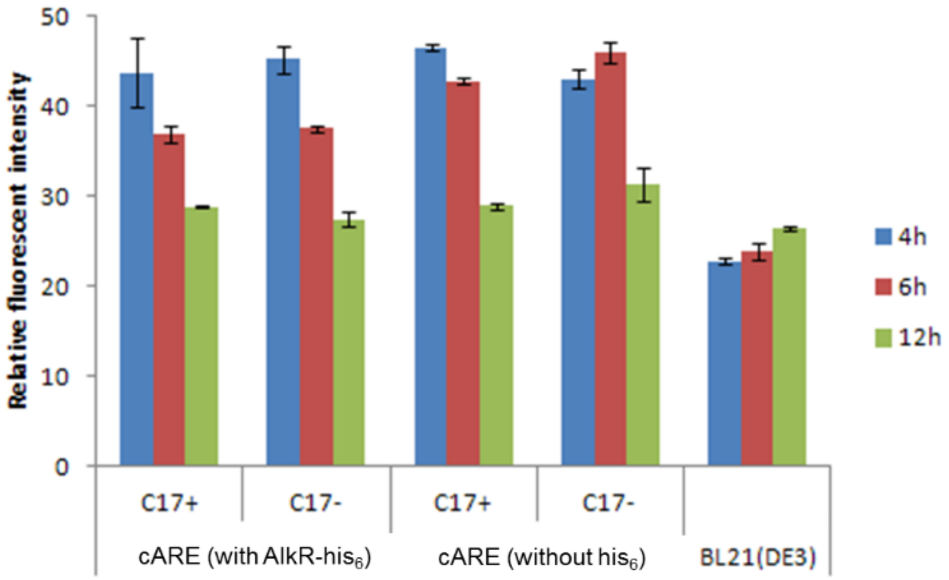

Supplementary Fig. 2丨Adding heptadecane (C17) from outside of the cells failed to induce the expression of GFP. In order to test the responsive ability of the biosensor to alkanes outside of the cell, 0.1 mM of C17 (dissolved in ethyl acetate) was added to the BL21(DE3) cells harboring the cARE biosensor at an OD_{600} of 0.5 in the modified mineral medium mentioned in the article. The cells were incubated at 30°C 250 rpm and sampled at 4 h, 6 h and 12 h after induction. The green fluorescence was read with a Synergy^{HT} fluorescence microplate reader at emission 475-495 nm, excitation 506-526 nm. Error bar gave the means of three independent cultures for each strain. No obvious diversity could be observed between the cells with or without C17.

Supplementary Fig. 3

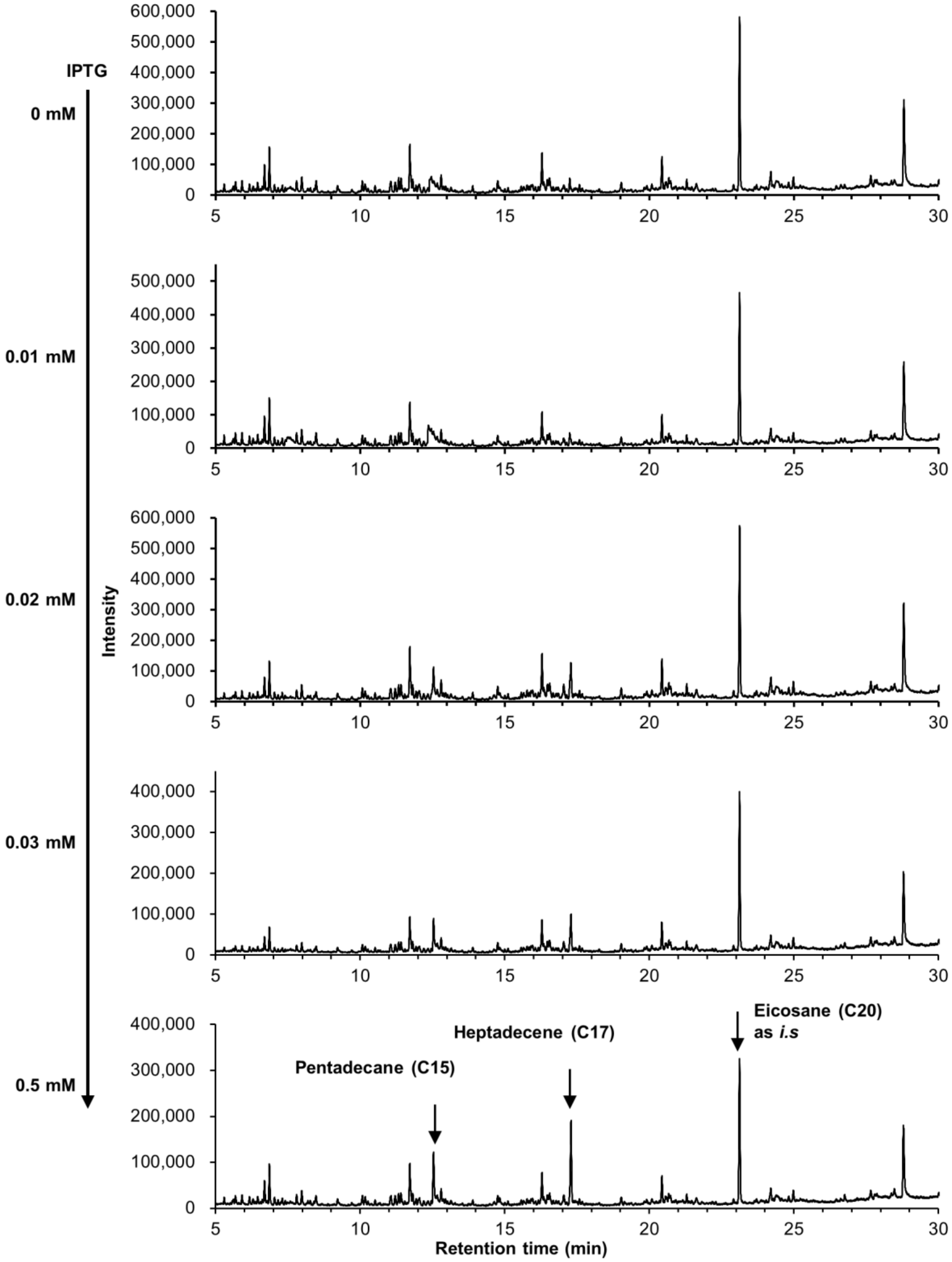

Supplementary Fig. 3│ Alkane yields in the recombinant alkane-producing *E. coli.* 10 mL of cells harboring both ASE and cARE were harvested 40 hours after induction, and extracted with 10 ml of chloroform–methanol (v/v, 2:1). 50 µg of n-eicosane (C20) was added as internal standards (*i.s.*)

before extraction. The total alkane yields were estimated according to the peak areas of the respective hydrocarbons that identified by MS analysis.

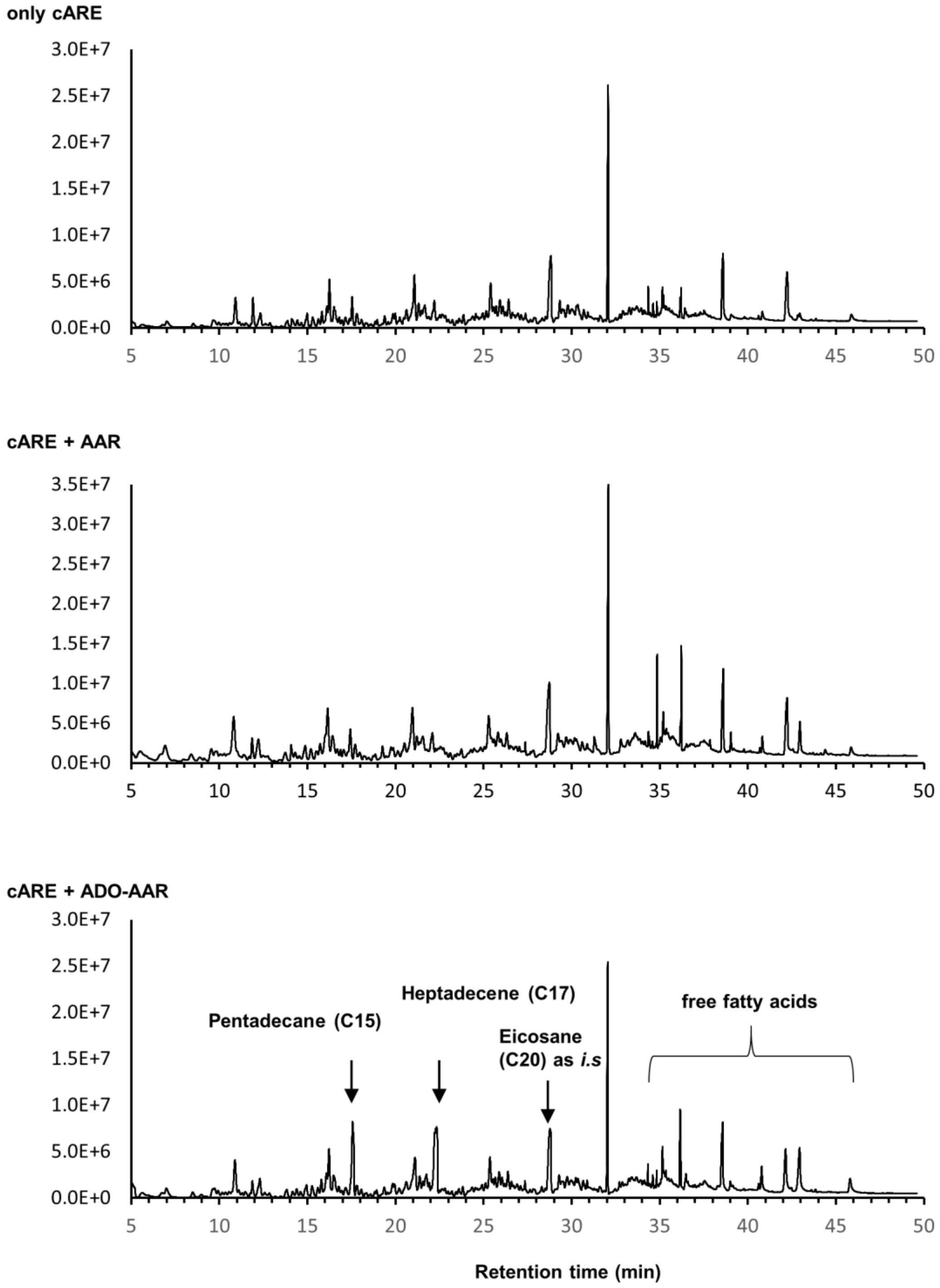

Supplementary Fig. 4│ Alkane yields in the recombinant *E. coli*. 10 mL of cells harboring either cARE or AAR+ADO-AAR or cARE+AAR were harvested 40 hours after induction, and extracted with 10 ml of chloroform–methanol (v/v, 2:1). 30 μg of n-eicosane (C20) was added as internal standards (*i.s.*) before extraction. As shown in the figure, no alkanes were detected in cells that without ADO.

Supplementary Table 1 Primers used in this study

Oligonucleotides	Sequences	Description
alkR-F	GCGAGAATAGCATATACATATGGATGCACTTAGTAAAAT	Forward primer for AlkR
alkR-R	ATCCCTCGAGTCATGATTGCTGGCGATAG	Reverse primer for AlkR
alkM-F	TCGCGGTACCTGGCATTCTAGAAAATGCCAAAGACTTTG	Forward primer for P_{alkM}
alkM-R	GCTCACCAT*GAATTC*AGTGAATCCTTTCTTGTTATCCTC	Reverse primer for P_{alkM}
alkS-F	GCCAGGTACCGCGAGCTACTCGCGAC	Forward primer for P_{alkS}
alkS-R	CATCCATATGTATATGCTATTCTCGCGCCAGCTGACT	Reverse primer for P_{alkS}
1593-F	ACGAGTACATATGCCGCAGCTTGAAGCCAGCCTTGAACT	Forward primer for ADO-AAR
1594-F	ACGAGTACATATGTTCGGTCTTATCGGTC	Forward primer for AAR
1594-R	CGAGCTCGAGTCAAATTGCCAATGCCAAG	Reverse primer for ADO-AAR
alkR-his-R	ACTCTCGAGTCAGTGGTGGTGGTGGTGGTGGCTGGATCCT GATTGCTGGCGATAGT	Reverse primer for AlkR-His_6
